# Pulmonary Vascular Resistance Measurement Remains Keystone in Congenital Heart Disease Management

**DOI:** 10.3389/fcvm.2021.607104

**Published:** 2021-03-31

**Authors:** Omar Tamimi, Mohammed H. A. Mohammed

**Affiliations:** ^1^Department of Cardiac Sciences, King Salman Cardiac Center, King Fahad Medical City, Riyadh, Saudi Arabia; ^2^Department of Cardiac Sciences, Ministry of National Guard Health Affairs, Riyadh, Saudi Arabia; ^3^King Abdullah International Medical Research Center (KAIMRC), Riyadh, Saudi Arabia; ^4^King Saud bin Abdulaziz University for Health Sciences, Riyadh, Saudi Arabia; ^5^Department Pediatric, Faculty of Medicine, Omdurman Islamic University, Omdurman, Sudan

**Keywords:** congenital, heart, cardiac, catheterization, resistance

## Abstract

Pulmonary vascular resistance (PVR) plays a major role in congenital heart management and critical decision. The impact of pulmonary vascular disease in the early and late morbidity and mortality after cardiac surgery and interventional catheterization in congenital heart defect (CHD) highlights the importance of critical evaluation for PVR. Currently, PVR is evaluated with invasive cardiac catheterization for hemodynamic data collection, processing, and analysis. Despite the limitation of hemodynamic evaluation in the setting of CHD, accurate data analysis, and interpretation have significant impact on clinical outcome and procedure success. This article reviews the basic calculation of PVR in the setting of congenital heart disease with diagrammatic illustration for easy understanding of the hemodynamic.

## Introduction

The pulmonary vascular resistance (PVR) is a good surrogate for pulmonary vascular disease at a certain level in patient with congenital heart disease. The surgical planning, risk of the procedure, and clinical decision are aligned with the presence or absence of significant pulmonary vascular disease (1). Although several clinical and noninvasive procedures give valuable data of hemodynamic condition of the patient with congenital heart defect (CHD), such as using Doppler echocardiography, which is a commonly implemented method for non-invasive measurement of pulmonary arterial pressure; however, Doppler echocardiography on the whole has a weakness in quantitative measurement because of limited acoustic window especially for evaluation of pulmonary circulation and the operator dependency for data acquisition (2, 3). In the current era, phase-contrast magnetic resonance imaging (MRI) is counted as an added non-invasive method for assessment of hemodynamics of pulmonary or systemic circulation (4, 5). These techniques offer the prospect for precise estimation of pulmonary circulation parameters, and their measurements were more accurate and reproducible than Doppler echocardiography (6, 7). Despite that nonetheless invasive right and left heart cardiac catheterization remains to be the gold-standard procedure of calculation PVR in the CHD field (8). The PVR calculation is based on the hydraulic version of Ohm's law (9). In this review, we will explain the hemodynamics of the cardiac lesion with diagrams showing the relation of different components of hemodynamics.

## Operability of Congenital Heart Disease

The current recommendation for operability in most of the guidelines for congenital heart disease with biventricular repair is PVR below 4–6 wood units per m^2^, whereas 4–8 wood units per m^2^ in gray zone need more clinical evaluation and case-based decision and >8 wood units per m^2^ nonoperable case (8–11).

## Hemodynamic Equation in Use for Vascular Resistant Evaluation

The major determinants of vascular resistance are transitional and muscular arterioles, which contain smooth muscle cell in their wall and allow for the vasoreactivity that serves to regulate pulmonary blood flow (12–14).

The PVR calculation is driven from the hydraulic version of Ohm's law: *I* = *V*/*R I* = current, *V* = voltage, *R* = resistance. *PVR* = *MPAP*−*LAP or PCWP*/*Qp*, Qp = pulmonary flow, MPAP = mean pulmonary artery pressure, PCWP = pulmonary capillary wedge pressure, LAP = mean left atrium pressure. Despite that the Ohm's law can be used for parallel or serial resistance, the complexity of congenital heart disease, which has a different flow and resistance, limits its uses for every lesion. Another consideration in operability is the amount of shunt and pulmonary flow, which uses modified Fick method and oxygen consumption tables (15–17). Qp: Qs = (arterial oxygen saturation—mixed venous oxygen saturation)/pulmonary venous oxygen saturation—pulmonary artery oxygen saturation Pulmonary flow (Qp) = oxygen consumption/Δ oxygen content difference of pulmonary venous and arterial blood.

## PVR in Normal Heart

**Figure 1:**
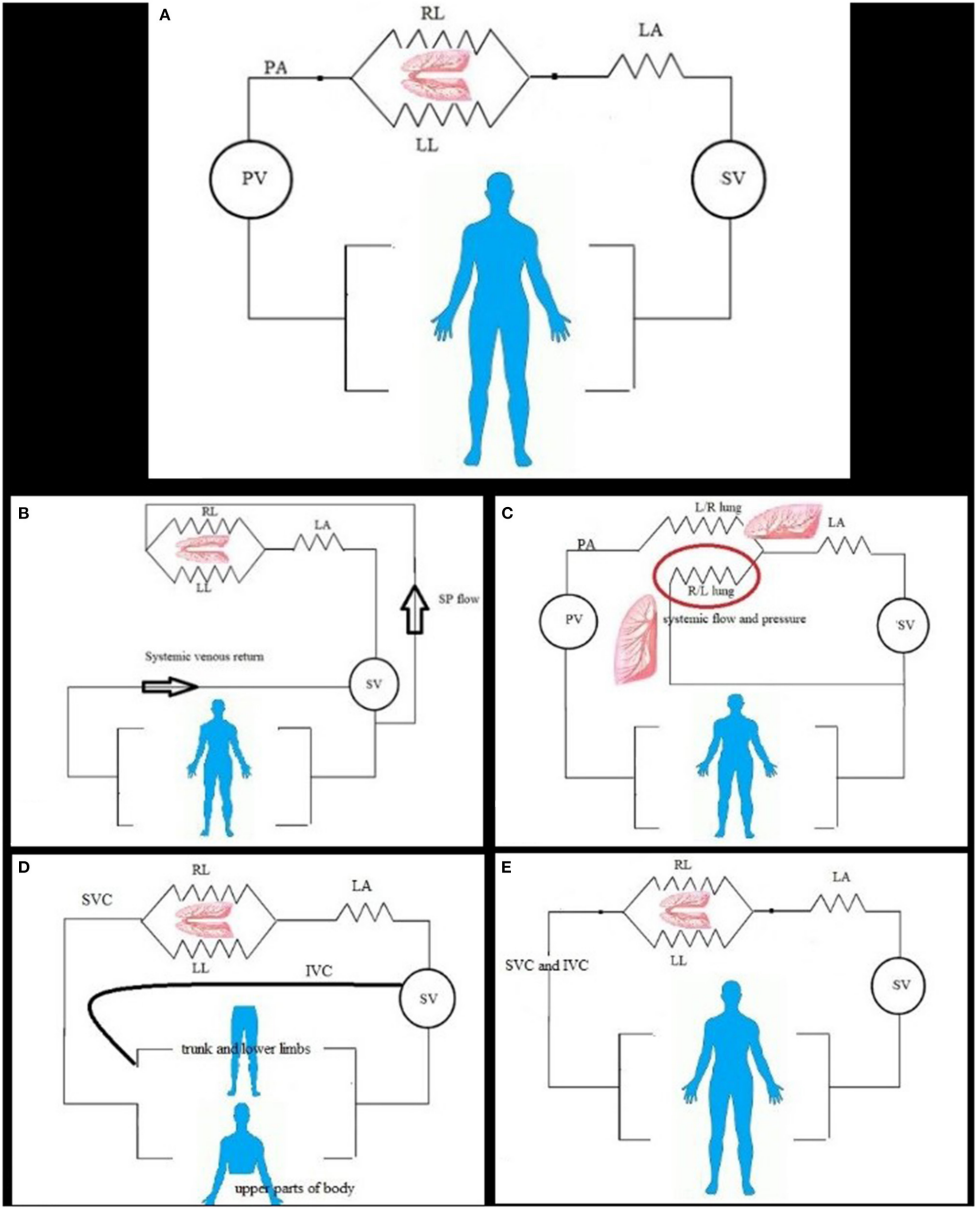
**(A)** In normal and simple shunt condition, the pulmonary flow is constant, and flow divided to the left (LL) and right (RL) lung, and then connected to the left atrium (LA); both lungs to the left atrium create flow resistance for calculation. Systemic (SV) and pulmonic (PV) ventricles work as pump and force generator. **(B)** In pulmonary flow from systemic, the pulmonary flow depends on the size of connection and systemic pressure and flow. The resistance can be calculated by direct measurement of pulmonary artery pressure and left atrium or wedge mean pressure and calculated pulmonary flow. The two lungs can be considered as one unit; no significant pulmonary artery stenosis and reduced flow to one lung. **(C)** Disconnected lung, the pulmonic ventricle (PV) ejects blood (pulmonary flow) to one lung (right or left lung R/L). The other lung is disconnected lung (red circle) and can have systemic blood supply from systemic ventricle (SV) via shunt or patent ductus arteriosus or major aortopulmonary collaterals and has parallel resistance with other systemic organs. **(D)** In the bidirectional cavopulmonary anastomosis, the upper part of the body venous return is the main blood supply for the lung; the PVR is calculated as in series resistance. The lower part of the body has venous return to the heart directly. **(E)** In total cavopulmonary anastomosis, the pulmonary artery is connected to systemic venous return and received total cardiac output flow.

The normal pulmonary flow unequally distributed to the left and right lungs, which qualify them for parallel resistance but are always considered as one unit, which will continue until we have a great, feasible tool to measure the flow of both lungs separately (Figure 1A). The cardiac MRI can be this tool, but until most of the issues related to expense, compatibility, and space occupation are resolved, the assumption of one unit will stay (14). Unlike the adult right heart catheterization, thermodilution used for cardiac output measurement in children is limited and replaced by modified Fick method with assumed oxygen consumption (15, 16). The indication for right heart catheterization with normal heart is most of the time related to pulmonary hypertension or preliver transplant.

## PVR With Simple Shunt

The simple cardiac shunt includes atrial and ventricular septal defect, partial anomalous pulmonary venous return, and patent ductus arteriosus, calculated with modified Fick method (15). The calculation with usual equation applies the guidelines for closure (Figure 1A).

## PVR With Exclusive Pulmonary Flow From the Aorta

When the congenital heart disease complex needs repair after a certain age, most of the time diagnostic cardiac catheterization with hemodynamic evaluation comes before the next stage. The pulmonary blood flow can be solely from the systemic blood in the following conditions: [1]. Pulmonary atresia with patent ductus or post–systemic-to-pulmonary shunt or major aortopulmonary collateral [2]. Truncus arteriosus [3]. Post–Norwood procedure stage 1 with systemic to pulmonary shunt In these cases, there is a complete mix of systemic and pulmonary venous blood at a ventricular level, so the pulmonary arterial oxygen saturation is equal to the systemic (arterial) oxygen saturation for flow and shunt calculation (Figure 1B). The PVR can be calculated in all by pressure measurement and flow calculation except for the lung with multiple blood supplies as major aortopulmonary collaterals where pulmonary pressure and differential segmental perfusion are significantly heterogeneous (17, 18). The hemodynamic results are determinate for the next surgical step as a single ventricle vs. biventricular repair.

## PVR With Single-Lung Supply by Systemic Shunt

In this setting of pathology, one lung taking the whole cardiac output and calculated resistance will be for that lung, whereas the second one supplied by systemic blood and exposed to systemic pressure needs to be calculated as separate flow and resistance. After surgical connection to the other lung, the flow will depend on the resistance at that lung and future evolution of pulmonary vascular changes (19) (Figure 1C). The PVR = mean PAP—mean LA pressure/flow to the connected lung Whereas, the disconnected lung has its own blood flow from arterial blood and systemic pressure, calculation of resistance by modified Fick method of oxygen consumption in this lung has a high rate of false error as the pulmonary artery and pulmonary venous oxygen saturation difference is minimal with overestimation of flow and underestimation of resistance (20). In such condition, cardiac MRI for the flow measurement of the disconnected lung gives accurate flow measurement and accurate resistance calculation (21).

## PVR With Cavopulmonary Anastomosis

The single-ventricle setting started with variable physiology with each stage of palliation. The usual first-stage palliation is maintaining adequate pulmonary flow by systemic-to-pulmonary shunt or control flow by pulmonary artery banding, whereas the second-stage palliation is bidirectional cavopulmonary anastomosis with significant flow reduction to the lung, as well as the volume load of the sub-systemic ventricle (22). The pulmonary blood flow depends on the venous return of the upper part of the body, which changes with the growth of body and decreases with time, without systemic augmentation of blood flow to the lung; the pulmonary blood flow is usually less than systemic flow (Qp:Qs <1) (23). In the current era, few studies showed that single-ventricle patients not requiring an intervention can undertake successful Fontan completion, depending on Cardiac MRI and echocardiography examinations only, with analogous short-term outcomes to those who underwent diagnostic catheterization, avoiding an invasive test, and exposure to radiation. Cardiac MRI can add information in a significant number of patients, it can be superior to catheterization in measuring aortopulmonary collaterals, but the catheterization definitely added more value in occlusions of venovenous collaterals that usually open after the second stage of a single ventricle palliation.

The final stage of palliation is total cavopulmonary anastomosis, and the pulmonary flow will be almost equal to systemic (Figures 1D,E). The success of the bidirectional anastomosis depends on low pulmonary resistance and mean pulmonary arterial pressure of <16 mmHg to have equilibrium between osmotic and hydrostatic pressure and low PVR of <2.5 w.u.m^2^ (24, 25). The calculation of PVR may underestimate the value as the cardiac output below the normal for age with absence of the subpulmonic ventricle (26).

## PVR Calculations in Parallel Circulations

As the calculation of the pulmonary resistance depends on the calculation of the pulmonary blood flow, which in most cases of the parallel circulation, such as transposition of great arteries, will not give an accurate result due to high pulmonary artery saturation, in such cases it is better to use other modalities such as cardiac MRI.

Another point that affects the vascular resistance calculation is in conditions when the small artery provides three smaller arterioles, each parallel to the other. The total resistance (Rx) for the three parallel arterioles comprising the segment would be as follows:

1/Rx = 1/R1 + 1/R2 +1/R3

R1 = resistance in arteriole 1, R2 = resistance in arteriole 2, R3 = resistance in arteriole 3

This proves the principles about the parallel arrangement of blood vessels:

The overall resistance of a network of parallel vessels is less than the resistance of the vessel having the lowest resistance. Once there are numerous parallel vessels, changing the resistance of a small number of these vessels will have a slight impact on the total resistance for the segment.

## Conclusion

The variable anatomic substrate of congenital heart disease and hemodynamic variation during clinical and invasive evaluation require a comprehensive understanding of all substrates of the blood circuit and appropriate calculation methods.

## Author Contributions

OT and MM contribute to idea, drawing, writing, and review equally.

## Conflict of Interest

The authors declare that the research was conducted in the absence of any commercial or financial relationships that could be construed as a potential conflict of interest.

## References

[1] Lopes AA Barst RJ Haworth SG Rabinovitch M Al Dabbagh M Del Cerro MJ. Repair of congenital heart disease with associated pulmonary hypertension in children: what are the minimal investigative procedures? Consensus statement from the congenital heart disease and pediatric task forces, pulmonary vascular research institute (PVRI). Pulmonary Circulat. (2014) 4:330–41. 10.1086/675995 25006452 PMC4070778

[2] Mansencal N Martin F Farcot JC Digne F Joseph T Pilliére R. Echocardiographic automated cardiac output measurement of pulmonary output and quantification of intracardiac shunt. Int J Cardiol. (2005) 104:25–31. 10.1016/j.ijcard.2004.09.012 16137505

[3] Cloez JL Schmidt KG Birk E Silverman NH. Determination of pulmonary to systemic blood flow ratio in children by a simplified Doppler echocardiographic method. J Am Coll Cardiol. (1988) 11:825–30. 10.1016/0735-1097(88)90218-5 3351150

[4] Mousseaux E Tasu JP Jolivet O Simonneau G Bittoun J Gaux JC. Pulmonary arterial resistance:noninvasive measurement with indexes of pulmonary flow estimated at velocity-encoded MR imaging–preliminary experience. Radiology. (1999) 212:896–902. 10.1148/radiology.212.3.r99au21896 10478263

[5] Lotz J Meier C Leppert A Galanski M. Cardiovascular flow measurement with phase-contrast MR imaging: basic facts and implementation. Radiographics. (2002) 22:651–71. 10.1148/radiographics.22.3.g02ma11651 12006694

[6] McDonnell CH III Herfkens RJ Norbash AM Rubin GD. Magnetic resonance imaging and measurement of blood flow. West J Med. (1994) 160:237–42. 8191756 PMC1022388

[7] Lee VS Spritzer CE Carroll BA Pool LG Bernstein MA Heinle SK. Flow quantification using fast cine phase-contrast MR imaging, conventional cine phase-contrast MR imaging, and Doppler sonography: *in vitro* and *in vivo* validation. AJR Am J Roentgenol. (1997) 169:1125–31. 10.2214/ajr.169.4.9308476 9308476

[8] Rosenzweig EB Abman SH Adatia I Beghetti M Bonnet D Haworth S. Paediatric pulmonary arterial hypertension: updates on definition, classification, diagnostics and management. ERJl. (2019) 53:1801916. 10.1183/13993003.01916-2018 30545978 PMC6351335

[9] Akers M Gassman RS. Hydraulic Power System Analysis. New York, NY: Taylor & Francis (2006). p. 299–329. 10.1201/9781420014587

[10] Galiè N Humbert M Vachiery JL Gibbs S Lang I Torbicki A. 2015 ESC/ERS guidelines for the diagnosis and treatment of pulmonary hypertension: the joint task force for the diagnosis and treatment of pulmonary hypertension of the European Society of Cardiology (ESC) and the European Respiratory Society. (ERS):Endorsed by: Association for European Paediatric and Congenital Cardiology. (AEPC), International Society for Heart and Lung Transplantation. (ISHLT). Eur Heart J. (2016) 37:67–119. 10.1093/eurheartj/ehv317 26320113

[11] Hansmann G Koestenberger M Alastalo TP Apitz C Austin ED Bonnet D. 2019 updated consensus statement on the diagnosis and treatment of pediatric pulmonary hypertension: the European Pediatric Pulmonary Vascular Disease Network (EPPVDN), endorsed by AEPC, ESPR and ISHLT. JHLT. (2019) 38:879–901. 10.1016/j.healun.2019.06.022 31495407

[12] Townsley MI. Structure and composition of pulmonary arteries, capillaries, and veins. Compr Physiol. (2012) 2:675–709. 10.1002/cphy.c100081 23606929 PMC3630377

[13] Rizzo AN Fraidenburg DR Yuan JXJ. Pulmonary vascular anatomy. In: Lanzer P, editors. PanVascular Medicine. Berlin: Springer (2015). p. 4041–56. 10.1007/978-3-642-37078-6_201

[14] Menon PG Adhypak SM Williams RB Doyle M Biederman RW. Investigating cardiac MRI based right ventricular contractility as a novel non-invasive metric of pulmonary arterial pressure. Clin Med Insights. Cardiol. (2015) 8(Suppl 1):45–50. 10.4137/CMC.S15711 25624777 PMC4285704

[15] Wilkinson JL. Haemodynamic calculations in the catheter laboratory. Heart. (2001) 85:113–20. 10.1136/heart.85.1.113 11119478 PMC1729580

[16] Lundell BP Casas ML Wallgren CG. Oxygen consumption in infants and children during heart catheterization. Pediatr Cardiol. (1996) 17:207–13. 10.1007/BF02524795 8662051

[17] Rudolph AM. Congenital Diseases of the Heart Clinical-Physiological Considerations, 3rd Edn. Chichester: Wiley-Blackwell (2009). p. 62–86. 10.1002/9781444311822

[18] Walley KR. Use of central venous oxygen saturation to guide therapy. Am J Respirat Crit Care Med. (2011) 184:514–20. 10.1164/rccm.201010-1584CI 21177882

[19] Egito ES Aiello VD Bosisio IB Lichtenfels AJ Horta AL Saldiva PH. Vascular remodeling process in reversibility of pulmonary arterial hypertension secondary to congenital heart disease. Pathol Res Pract. (2003) 199:521–32. 10.1078/0344-0338-00457 14533936

[20] Fritts HW Cournand A. The application of the fick principle to the measurement of pulmonary blood flow. Proc Natl Acad Sci USA. (1958) 44:1079–87. 10.1073/pnas.44.10.1079 16590311 PMC528697

[21] Körperich H Gieseke J Barth P Hoogeveen R Esdorn H Peterschröder A. Flow volume and shunt quantification in pediatric congenital heart disease by real-time magnetic resonance velocity mapping:a validation study. Circulation. (2004) 109:1987–93. 10.1161/01.CIR.0000126494.66859.A2 15066942

[22] Gewillig M Brown SC. The Fontan circulation after 45 years: update in physiology. Heart. (2016) 102:1081–6. 10.1136/heartjnl-2015-307467 27220691 PMC4941188

[23] Kondpalle S Lote-Oke R Patel P Khadilkar V Khadilkar AV. Upper and lower body segment ratios from birth to 18 years in children from Western Maharashtra. Ind J Pediatr. (2019) 86:503–7. 10.1007/s12098-019-02883-x 30756289

[24] Aukland K. Distribution of body fluids: local mechanisms guarding interstitial fluid volume. J Physio. (1984) 79:395–400. 6399307

[25] Gewillig M. The Fontan circulation. Heart. (2005) 91:839–46. 10.1136/hrt.2004.051789 15894794 PMC1768934

[26] Rychik J Atz AM Celermajer DS Deal BJ Gatzoulis MA Gewillig MH. American heart association council on cardiovascular disease in the young and council on cardiovascular and stroke nursing. Evaluation and Management of the Child and Adult with Fontan Circulation: a scientific statement from the American Heart Association. Circulation. (2019) 2019:CIR0000000000000696. 10.1161/cir.0000000000000696 31256636

